# When should we start renal-replacement therapy in critically ill patients with acute kidney injury: do we finally have the answer?

**DOI:** 10.1186/s13054-021-03600-x

**Published:** 2021-05-26

**Authors:** Sean M. Bagshaw, Eric A. Hoste, Ron Wald

**Affiliations:** 1 Department of Critical Care Medicine, Faculty of Medicine and Dentistry, University of Alberta and Alberta Health Services, 2-124E Clinical Science Building, 8440-112 Street NW, Edmonton, AB T6G2B7 Canada; 2 Intensive Care Unit, Ghent University Hospital, Ghent, Belgium; 3 Division of Nephrology, St. Michael’s Hospital and the University of Toronto and the Li Ka Shing Knowledge Institute of St. Michael’s Hospital, 30 Bond Street, Toronto, ON M5B 1W8 Canada

## Background

A significant proportion of critically ill patients with severe AKI, particularly those who develop refractory complications [1], receive support with renal-replacement therapy (RRT) [2]. There has been a longstanding dilemma on when RRT should be started for patients with severe AKI, specifically among those without AKI-related complications that could be addressed by RRT. Among patients with urgent or refractory complications, there is consensus for starting RRT. However, should RRT be started earlier in the course of AKI to pre-empt complications or judiciously delayed and started if and when complications arise? Furthermore, clinicians are challenged to select those patients who will have high probability of clinical benefit (restore metabolic/fluid homeostasis) and improved outcome (survival, recovery, quality of life) and to avoid RRT in patients who do not need it, will not benefit from it or who have greater probability of experiencing harm from it.

Several randomized trials have shown that an earlier strategy for starting RRT does not confer a survival advantage, can increase the risk of harm (dialysis dependence [3], bacteremia [4]) and portend greater resource use compared with a “watch-and-wait” or delayed strategy [3–5]. These trials now support the broad notion that deferral of RRT, pending the development of urgent indications, should be adopted as the default strategy. The rationale for a “watch-and-wait” approach is to provide opportunity to observe for recovery and to avoid RRT in selected patients [3–5]. However, the variables that define “deferral” remain uncertain. Specifically, when confronted with patients who have severe and non-resolving AKI, for how long can deferral be considered safe and acceptable? This was addressed by the recent Artificial Kidney Initiation in Kidney Injury-2 (AKIKI-2) trial [6].

AKIKI-2 was a multi-centre trial comparing two strategies for delayed RRT initiation in 278 patients. Similar to AKIKI, patients fulfilled criteria for Stage 3 AKI and were receiving either mechanical ventilation and/or vasopressors. However, where AKIKI-2 differed is that eligibility specified a protracted AKI course, defined by oligo-anuria for ≥ 72 h and/or a serum urea 40–50 mmol/L (112–140 md/dL). Upon fulfilling these criteria, patients were randomized to “*delayed*” RRT (aligned with the delayed strategy of AKIKI [4]) started within 12 h of randomization or “*more-delayed*” RRT, defined by deferring RRT until an urgent indication emerged or serum urea exceeded 50 mmol/L (140 md/dL). Oligo-anuria was not a trigger for RRT in the more-delayed strategy. The primary outcome was “days-alive and RRT-free” from randomization through 28 days, conditional on patients being alive and RRT free for 3-consecutive days.

RRT-free days did not differ between the strategies. There were also no differences in secondary endpoints, including ventilator-free days, ICU stay or kidney recovery. In a pre-specified analysis, allocation to the more-delayed strategy was found to increase the hazard of death (adjusted-HR 1.65) compared with the delayed strategy.

Inherent in the AKIKI-2 design, it was hypothesized that the more-delayed strategy would increase in RRT-free days. It is therefore notable that RRT-free days were not greater with the more-delayed strategy, despite fewer patients receiving RRT and when initiated, occurring ~ 2 days later, when compared with the delayed strategy. As such, the likely driver of the primary outcome was higher mortality with the more-delayed strategy. This could have plausibly been driven by several factors, such as the effects of prolonged untreated AKI, exaggerated non-renal organ dysfunction (e.g., delirium [7]) and modified recovery from critical illness [8]. The protocol embedded precautions to prevent intra-dialytic hypotension and dialysis-disequilibrium syndrome, but these events were not reported. Finally, small and unblinded trials would be susceptible to biased co-interventions, such as withdrawal of life-sustaining treatments.

AKIKI-2 is applicable to a small proportion of patients with AKI. Of the 5,336 patients screened, only 767 (14.4%) fulfilled the initial eligibility. Of the 4,466 initially excluded, 42.9% failed to achieve Stage 3 AKI and many were excluded for urgent indications (13.5%) or prior RRT (6.8%). Moreover, of those with Stage 3 AKI who were excluded, 127 (26.0%) developed an urgent indication after a median (IQR) of 35 (17–68) hours and received RRT, while the remainder did not fulfil randomization criteria.

Similar to AKIKI, AKIKI-2 again suggests that serum urea as a primary trigger is not ideal for identification of the optimal timing of RRT, regardless of the thresholds evaluated [9]. First, serum urea was not useful for identifying patients with an increased mortality risk [10]. We currently lack robust data on serum urea thresholds that constitute toxicity. Rather than a specific threshold, the duration of elevated serum urea may be a better surrogate for the development of uremic-related complications. Second, serum urea is influenced by a range of factors other than impaired excretion, including excess protein catabolism, corticosteroid administration, exogenous protein or gastrointestinal bleeding and volume contraction. Finally, the thresholds of serum urea did not discriminate patients with urgent indications for RRT: 16.6% of Stage 3 AKI patients had urgent indications developed prior to the delayed criteria being fulfilled, and 33% of patients in the more-delayed strategy developed urgent indications prior to the protocolized serum urea threshold (> 50 mmol/L) was fulfilled.

AKIKI-2 highlights the need for robust and validated tools that discriminate between patients with AKI who are most likely to need and benefit from starting RRT from those in whom it can or should be avoided. Such tools include clinical prediction by machine learning [11], predictive enrichment with AKI sub-phenotypes, use of the furosemide stress test and emerging biomarkers of persistent severe AKI [12].

## Conclusions

**Fig. 1:**
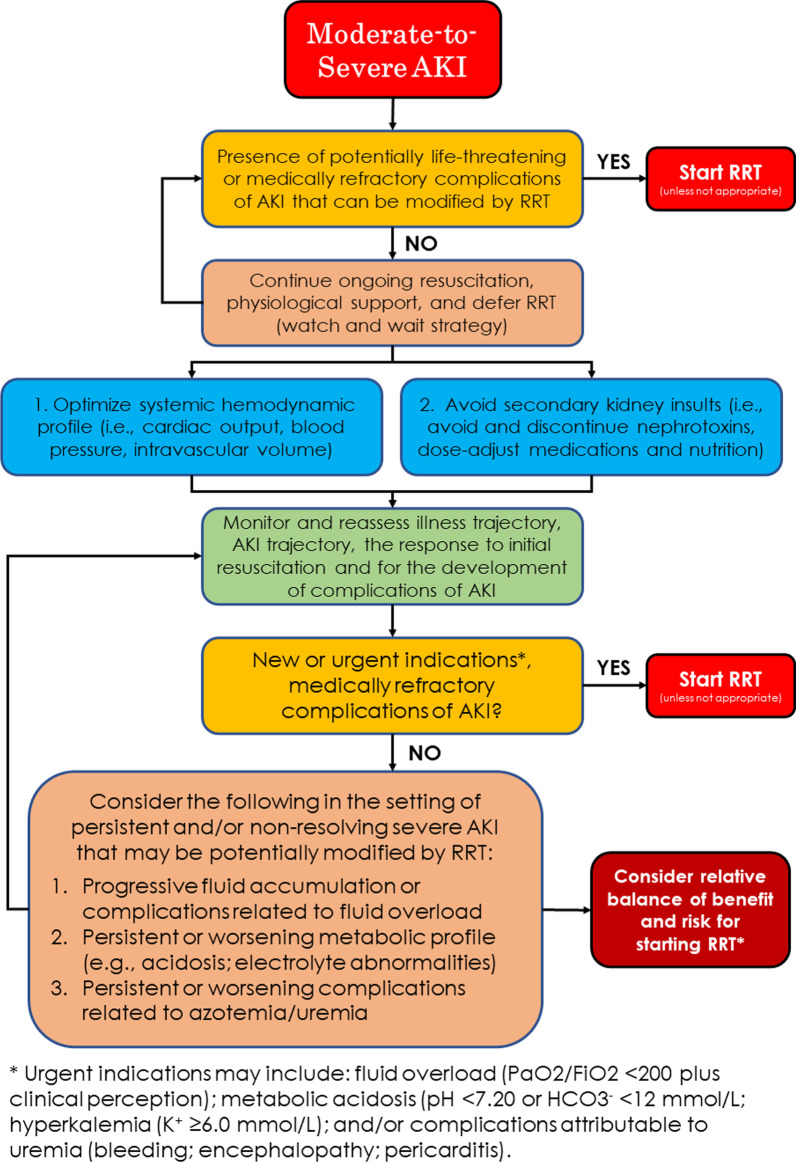
Proposed algorithm for starting renal-replacement therapy for critically ill patients with moderate-to-severe acute kidney injury (adapted from [13, 14]). *Urgent indications include fluid overload (PaO2/FiO2 < 200 plus clinical perception); metabolic acidosis (pH < 7.20 or HCO3^−^ < 12 mmol/L; hyperkalemia (K^+^  ≥ 6.0 mmol/L); and/or complications attributable to uremia (bleeding; encephalopathy; pericarditis)

Recent evidence has shown that pre-emptive or earlier RRT in patients with severe AKI and no urgent indications does not confer clinical benefit. By default, this would imply that a more judicious “watch and wait” strategy is acceptable. The findings of AKIKI-2 reinforce that there are limitations and harm to protracted delays in RRT initiation in patients with severe and persistent AKI [6]. Clinicians are bound to remain challenged by a lack of clarity on the optional circumstances to initiate RRT until additional evidence emerges. In the meantime, such uncertainty should not negate the importance of sound patient-centred practice grounded in the available evidence (Fig. 1). Clinicians should integrate an individual patient’s evolving critically illness, their dynamic response to interventions, the trajectory of AKI and likelihood for recovery, and importantly, patient and family preferences for care.
